# Relationship between arm-to-leg and limbs-to-trunk body composition ratio and cardiovascular disease risk factors

**DOI:** 10.1038/s41598-021-96874-8

**Published:** 2021-08-31

**Authors:** Sunmi Jung, Jihyun Park, Young-Gyun Seo

**Affiliations:** Department of Family Medicine, Hallym University Sacred Heart Hospital, 22, Gwanpyeong-ro 170beon-gil, Dongan-gu, Anyang-si, Gyeonggi-do 14068 Republic of Korea

**Keywords:** Cardiovascular diseases, Metabolic syndrome, Obesity

## Abstract

We aimed to analyze the relationship of the distribution of body fat mass (FM) and fat-free mass (FFM) in the limbs and trunk with the prevalence of cardiovascular disease risk factors (CVD-RF). In total, 13,032 adults were selected from the KNHANES (2008–2011). The prevalence of hypertension, diabetes mellitus (DM), dyslipidemia, and metabolic syndrome (MetS) according to the arm-to-leg ratio and limbs-to-trunk ratio for FM and FFM was compared, respectively. The higher the arm-to-leg FM ratio, the higher the prevalence of CVD-RF (DM-male-OR 7.04, 95% CI 4.22–11.74; DM-female-OR 10.57, 95% CI 5.80–19.26; MetS-male-OR 4.47, 95% CI 3.41- 5.86; MetS-female-OR 8.73, 95% CI 6.38–11.95). The higher the limbs-to-trunk FM ratio (DM-male-OR 0.12, 95% CI 0.07–0.21; DM-female-OR 0.12, 95% CI 0.06–0.23; MetS-male-OR 0.06, 95% CI 0.04–0.08; MetS-female-OR 0.02, 95% CI 0.01–0.04), the higher the limbs-to-trunk FFM ratio (DM-male-OR 0.19, 95% CI 0.11–0.31; DM-female-OR 0.46, 95% CI 0.30–0.70; MetS-male-OR 0.39, 95% CI 0.31–0.50; MetS-female-OR 0.62, 95% CI 0.50–0.78), and the higher the arm-to-leg FFM ratio (MetS-male-OR 0.75, 95% CI 0.59–0.94; MetS-female-OR 0.73, 95% CI 0.58–0.92), the lower the prevalence of CVD-RF. The higher the FM of the legs compared to the arms, FFM of the arms compared to the legs, and FM or FFM of the limbs compared to the trunk, the lower the prevalence of CVD-RF.

## Introduction

The prevalence of cardiovascular disease (CVD) and metabolic syndrome (MetS) is increasing worldwide^1,2^. MetS is a chronic metabolic disorder that involves a combination of CVD risk factors (CVD-RF) including high triglyceride (TG) levels, low high-density lipoprotein cholesterol (HDL-C) levels, high blood pressure (BP), and impaired fasting plasma glucose (FPG)^3,4^, and it is known to cause diabetes mellitus (DM) and other CVDs^5^.

Obesity, which is defined as excessive accumulation of body fat mass (FM), increases the risk of MetS. In particular, abdominal obesity increases the risk of CVD by inducing insulin resistance^6^, but femoral fat is related to buffering or improving the adverse effects of visceral fat cells rather than metabolic abnormalities^7^.

Muscle is a major organ that participates in glucose metabolism. Sarcopenia, or low muscle mass, is associated with reduced physical activity and energy imbalance and causes MetS by reducing the utilization of glucose and free fatty acid^8^. Sarcopenic obesity, which involves both obesity and low muscle mass, is known to lead to a higher risk of insulin resistance, DM, and MetS than that with obesity alone^9^.

Studies related to increased FM or sarcopenic obesity mainly analyzed the relationship between total FM or total fat-free mass (FFM) and the risk of CVD^10,11^. According to studies about regional FM, femoral fat was effective in improving glycemic control and lipid metabolism^12^ and lowering the risk of MetS and CVD^13,14^. In elderly women, peripheral FM showed an independent anti-atherogenic effect^15^, and central FM showed a positive correlation with arterial stiffness, whereas peripheral FM had a negative correlation^16^. In regional FFM-related studies, one study of DM patients with obesity reported that relatively preserved leg muscle mass is associated with improved MetS parameters such as body mass index (BMI), waist-to-hip ratio, waist circumference (WC), and HDL-C^17^. Another study of postmenopausal women described that FFM in the arm is associated with prevalence and severity of MetS^18^.

As previously reported, the risk of CVD varies depending on the distribution of body composition. Therefore, it is important to understand the relationship between the regional FM or FFM distribution and the CVD-RF. In addition, there may be an imbalance in FM or FFM between the arms and legs or between the limbs and trunk, depending on factors such as sex, age, and physical activity. Therefore, it is necessary to consider these factors. However, there are only few studies on the relationship of regional FM or FFM ratios and the risk of CVD. This study aimed to analyze the association between FM or FFM ratios of the arms, legs, and trunk and the prevalence of CVD-RF including hypertension (HTN), DM, dyslipidemia (DL), and MetS using the Korea National Health and Nutrition Examination Survey (KNHANES) data.

## Subjects and methods

### Study population

This study obtained data from the 4th and 5th KNHANES (2008–2011), including whole‐body dual-energy X‐ray absorptiometry (DEXA). Of a total of 18,915 subjects, those aged ≥ 19 years were included in the analysis. We excluded those who had severe decline in kidney function (estimated glomerular filtration rate < 30 mL/min/1.73 m^2^), a history of cancer diagnosis, inappropriate fasting duration before examination (> 24 h or < 8 h), inappropriate nutritional intake (< 500 kcal/day or > 5000 kcal/day), inappropriate water intake per body weight (≥ 90 g/kg), and missing data in questionnaire records or unavailable test results (5883 persons). Finally, a total of 13,032 eligible participants (5425 men and 7607 women) were included in the analysis (Supplementary Fig. S1).

All procedures were approved by the ethics committee of the Korea Disease Control and Prevention Agency (Approval Number: 2011-02CON-06-C, 2010-02CON-21-C, 2009-01CON-03-2C, and 2008-04EXP-01-C), and the study was carried out in accordance with the ethical standards laid down in the 1964 Declaration of Helsinki and its later amendments. Signed informed consent was obtained from all KNHANES participants. The KNHANES data are publicly available.

### Assessment of body compositions

Body compositions were measured using DEXA (Hologic Discovery, Hologic, USA)^19,20^, including regional FM (both arms, both legs, and trunk), FFM (both arms, both legs, and trunk), and bone mineral contents (BMC) (both arms, both legs, right ribs, left ribs, thoracic and lumbar vertebrae, pelvis), to obtain data. Using these data, regional FM and FFM were calculated; FM of arms is the sum of FM in both arms, FM of legs is the sum of FM in both legs, FFM of arms is the value excluding both arm BMC from the sum of FFM in both arms; FFM of legs is the value excluding both leg BMC from the sum of FFM in both legs, and FFM of trunk is the value excluding the right ribs, left ribs, thoracic and lumbar vertebral, and pelvic BMC from the trunk FFM. Therefore, in this study, FFM is not exactly muscle mass and may include water and other organs as well as muscle mass. The arm-to-leg ratio or limbs-to-trunk ratio was calculated for FM and FFM, respectively, and grouped into quartiles from the lowest value (Q1) to the highest value (Q4).

### Definitions of MetS

MetS was defined using two guidelines. First, based on the 2001 National Cholesterol Education Program (NCEP)/Adult Treatment Panel III (ATP III)^3^ and 2005 American Heart Association (AHA)/National Heart, Lung, and Blood Institute (NHLBI)^21^, MetS was defined when three or more of the following five criteria were satisfied: (1) WC ≥ 90 cm in men or ≥ 85 cm in women (based on abdominal obesity in Korean adults^22^); (2) TG ≥ 150 mg/dL or currently taking anti-dyslipidemic medications; (3) HDL-C < 40 mg/dL in men or < 50 mg/dL in women; (4) BP ≥ 130/85 mmHg or currently taking anti-hypertensive medications; (5) FPG ≥ 100 mg/dL or currently taking anti-hyperglycemic medications. Second, in accordance with the guideline of the 2006 International Diabetes Federation (IDF)^4^, MetS was defined when the abdominal obesity criterion (WC ≥ 90 cm in men or ≥ 85 cm in women) was met, and two or more of the following four criteria were satisfied: (1) TG ≥ 150 mg/dL or currently taking anti-dyslipidemic medications; (2) HDL-C < 40 mg/dL in men or < 50 mg/dL in women; (3) systolic BP ≥ 130 mmHg, diastolic BP ≥ 85 mmHg, or currently taking anti-hypertensive medications; (4) FPG ≥ 100 mg/dL or previous diagnosis of type 2 DM.

### Measurement of covariates

The general characteristics of the participants by sex were analyzed using data on age, BMI, WC, FM, FFM, daily energy intake (total energy intake (TEI) and water intake per body weight (WIBW)), smoking (never, past, or current), alcohol drinking (< once or ≥ once/month), education (≤ elementary school, middle or high school, or ≥ college), average monthly household income (AMHI) (quartile), previous diagnosis of HTN by doctors, previous diagnosis of DM by doctors, previous diagnosis of DL by doctors, and survey year. Physical activity (PA) was described as Metabolic Equivalent (MET) or classified as low, moderate, or high PA level based on the data processing and analysis guidelines of the International Physical Activity Questionnaire (IPAQ)^23^.

### Statistical analysis

Statistical analysis was conducted using the STATA, ver. 14.0, statistical program, and statistical significance level was defined as *p* < 0.05. The sampling design for the KNHANES used two-stage stratified cluster sampling, not simple random sampling. For this reason, when analyzing data, weights were applied by reflecting the contents of this complex sampling design. Linear regression analyses and chi-square tests were used in comparative analysis of general characteristics by sex. The chi-square tests and the logistic regression analyses were performed to compare the prevalence of HTN, DM, DL and MetS according to the arm-to-leg ratio or the limbs-to-trunk ratio for FM and FFM, respectively. Odds ratio (OR) was calculated by adjusting for age, TEI, WIBW, smoking, alcohol drinking, PA, education, AMHI, and survey year. A linear regression was then conducted by regressing the OR to evaluate the trend (*p* for trend). In addition, linear regression analyses and chi-square tests were used in the analysis of FM or FFM ratio between body parts and the regional fat-to-fat-free mass ratio according to age and BMI.

## Results

### General characteristics of the study population by sex

The general characteristics of the study population by sex are shown in Table 1. Mean age of 13,032 participants was 44.22 years old, and 58.37% were women. The mean arm-to-leg FM ratio of all participants was 0.37, the mean limbs-to-trunk FM ratio was 0.87, the mean arm-to-leg FFM ratio was 0.32, and the mean limbs-to-trunk FFM ratio was 0.86. The arm-to-leg FM ratio (0.37 ± 0.002 vs. 0.36 ± 0.002), arm-to-leg FFM ratio (0.34 ± 0.001 vs. 0.29 ± 0.001), and limbs-to-trunk FFM ratio (0.91 ± 0.001 vs. 0.80 ± 0.001) were higher in men than in women, and the limbs-to-trunk FM ratio (0.75 ± 0.004 vs. 0.99 ± 0.005) was lower in men than in women. WC, BMI, TEI, and WIBW were higher in men than in women; proportion of current smokers, alcohol drinkers (≥ once/month), participants with a high PA level, highly educated participants (college level or more), previous diagnosis of HTN or DM by doctors and MetS were also higher in men than in women. The proportion of previous diagnosis of DL by doctors was higher in women than in men.

**Table 1:** General characteristics of the study participants.

Characteristics	Total (n = 13,032)	Male (n = 5425)	Female (n = 7607)	*P* value^a^
Age, years	44.22 ± 0.26	44.03 ± 0.33	44.40 ± 0.28	0.247
Arm-to-leg fat mass ratio	0.37 ± 0.001	0.37 ± 0.002	0.36 ± 0.002	< 0.001
Limbs-to-trunk fat mass ratio	0.87 ± 0.004	0.75 ± 0.004	0.99 ± 0.005	< 0.001
Arm-to-leg fat-free mass ratio	0.32 ± 0.001	0.34 ± 0.001	0.29 ± 0.001	< 0.001
Limbs-to-trunk fat-free mass ratio	0.86 ± 0.001	0.91 ± 0.001	0.80 ± 0.001	< 0.001
Waist circumference, cm	80.67 ± 0.14	84.98 ± 0.18	77.62 ± 0.18	< 0.001
Body mass index, kg/m^2^	23.59 ± 0.04	24.04 ± 0.06	23.19 ± 0.06	< 0.001
**Nutritional intake**				
Total energy, kcal/day	1988.60 ± 10.37	2331.98 ± 15.05	1673.49 ± 9.70	< 0.001
Water intake/body weight, g/kg/day	15.73 ± 0.13	16.41 ± 0.17	15.11 ± 0.16	< 0.001
**Smoking**				< 0.001
None	8176 (62.74)	1219 (22.47)	6957 (91.46)	
Past	2273 (17.44)	1989 (36.66)	284 (3.73)	
Current	2583 (19.82)	2217 (40.87)	366 (4.81)	
**Alcohol consumption**				< 0.001
< 1 time/month	5945 (45.62)	1377 (25.38)	4568 (60.05)	
≥ 1 time/month	7087 (54.38)	4048 (74.62)	3039 (39.95)	
**Physical activity (IPAQ)**^**b**^				< 0.001
Low	3990 (30.62)	1501 (27.67)	2489 (32.72)	
Moderate	5368 (41.19)	2101 (38.73)	3267 (42.95)	
High	3674 (28.19)	1823 (33.60)	1851 (24.33)	
**Education**				< 0.001
≤ Elementary school	3009 (23.09)	860 (15.85)	2149 (28.25)	
Middle or high school	5349 (41.05)	2278 (41.99)	3071 (40.37)	
≥ College	4674 (35.87)	2287 (42.16)	2387 (31.38)	
**Income**				0.560
Q1	3121 (23.95)	1277 (23.54)	1844 (24.24)	
Q2	3367 (25.84)	1413 (26.05)	1954 (25.69)	
Q3	3313 (25.42)	1363 (25.12)	1950 (25.63)	
Q4	3231 (24.79)	1372 (25.29)	1859 (24.44)	
**Survey year**				0.050
2008	2400 (18.42)	940 (17.33)	1460 (19.19)	
2009	5021 (38.53)	2127 (39.21)	2894 (38.04)	
2010	3976 (30.51)	1681 (30.99)	2295 (30.17)	
2011	1635 (12.55)	677 (12.48)	958 (12.59)	
**Comorbidity**				
Hypertension	2648 (20.32)	1209 (22.29)	1439 (18.92)	< 0.001
Diabetes	940 (7.21)	457 (8.42)	483 (6.35)	< 0.001
Dyslipidemia	1178 (9.04)	445 (8.20)	733 (9.64)	0.005
**MetS component**				
WC ≥ 90 cm	3330 (25.55)	1428 (26.32)	1902 (25.00)	0.089
TG ≥ 150 mg/dL or drug treatment for dyslipidemia	3559 (27.31)	1994 (36.76)	1565 (20.57)	< 0.001
HDL-C < 40 mg/dL	5766 (44.24)	1830 (33.73)	3936 (51.74)	< 0.001
BP ≥ 130/85 mmHg or drug treatment for elevated BP	3624 (27.81)	1807 (33.31)	1817 (23.89)	< 0.001
FPG ≥ 100 mg/dL or drug treatment for elevated FPG	3447 (26.45)	1798 (33.14)	1649 (21.68)	< 0.001
MetS (NCEP)	3070 (23.56)	1435 (26.45)	1635 (21.49)	< 0.001
MetS (IDF)	2071 (15.89)	915 (16.87)	1156 (15.20)	0.010

Data are presented as mean ± SE for continuous variables and numbers (%) for categorical variables.

IPAQ, International Physical Activity Questionnaire; MetS, metabolic syndrome; WC, waist circumference; TG, triglyceride; HDL-C, high-density lipoprotein cholesterol; BP, blood pressure; FPG, fasting plasma glucose; NCEP, 2005 National Cholesterol Education Program Adult Treatment Panel III; IDF, 2006 International Diabetes Federation.

^a^*P* value from linear regression analysis for continuous variables or χ^2^ test for categorical variables, comparing differences between two groups.

^b^Categorical variable from the International Physical Activity Questionnaire research committee.

### Body composition distribution and prevalence of cardiovascular disease risk factors

A comparison of the prevalence of CVD-RF according to the distribution of body composition is shown in Table 2. In the arm-to-leg FM ratio, the prevalence of HTN, DM, DL, and MetS was the highest in Q4 in both men and women, and it increased as the ratio increased. In the limbs-to-trunk FM ratio, the prevalence of HTN, DM, DL, and MetS was the highest in Q1 in both men and women, and it increased as the ratio decreased. In the arm-to-leg FFM ratio, the prevalence of HTN, DM, DL, and MetS was the highest in Q4 in women, but DL was the highest in Q2 and MetS (IDF) was the highest in Q1 in men. In the limbs-to-trunk FFM ratio, the prevalence of HTN, DM, DL, and MetS was the highest in Q1 in both men and women, and it increased as the ratio decreased.

**Table 2:** Prevalence of cardiovascular disease risk factors according to body composition ratio quartile.

Male Arm-to-leg fat mass ratio	Q1 (n = 1322)	Q2 (n = 1358)	Q3 (n = 1370)	Q4 (n = 1375)	*P* value^a^
Hypertension	132 (9.98)	278 (20.47)	374 (27.30)	425 (30.91)	< 0.001
Diabetes	26 (1.97)	82 (6.04)	123 (8.98)	226 (16.44)	< 0.001
Dyslipidemia	46 (3.48)	100 (7.36)	132 (9.64)	167 (12.15)	< 0.001
MetS (NCEP)	138 (10.44)	321 (23.64)	442 (32.26)	534 (38.84)	< 0.001
MetS (IDF)	92 (6.96)	207 (15.24)	294 (21.46)	322 (23.42)	< 0.001

Female Arm-to-leg fat mass ratio	Q1 (n = 1828)	Q2 (n = 1902)	Q3 (n = 1956)	Q4 (n = 1921)	*P* value^a^
Hypertension	94 (4.89)	230 (12.05)	438 (22.96)	677 (36.22)	< 0.001
Diabetes	14 (0.73)	44 (2.30)	115 (6.03)	310 (16.59)	< 0.001
Dyslipidemia	36 (1.87)	132 (6.91)	229 (12.00)	336 (17.98)	< 0.001
MetS (NCEP)	82 (4.27)	224 (11.73)	495 (25.94)	834 (44.62)	< 0.001
MetS (IDF)	47 (2.45)	144 (7.54)	352 (18.45)	613 (32.80)	< 0.001

Male Limbs-to-trunk fat mass ratio	Q1 (n = 1322)	Q2 (n = 1358)	Q3 (n = 1370)	Q4 (n = 1375)	*P* value^a^
Hypertension	514 (37.82)	365 (26.35)	211 (15.47)	119 (9.04)	< 0.001
Diabetes	245 (18.03)	126 (9.10)	60 (4.40)	26 (1.97)	< 0.001
Dyslipidemia	204 (15.01)	138 (9.96)	73 (5.35)	30 (2.28)	< 0.001
MetS (NCEP)	660 (48.57)	457 (33.00)	257 (18.84)	61 (4.63)	< 0.001
MetS (IDF)	447 (32.89)	294 (21.23)	148 (10.85)	26 (1.97)	< 0.001

Female Limbs-to-trunk fat mass ratio	Q1 (n = 1828)	Q2 (n = 1902)	Q3 (n = 1956)	Q4 (n = 1921)	*P* value^a^
Hypertension	747 (40.44)	461 (24.80)	187 (9.61)	44 (2.25)	< 0.001
Diabetes	293 (15.86)	127 (6.83)	49 (2.52)	14 (0.72)	< 0.001
Dyslipidemia	358 (19.38)	220 (11.83)	116 (5.96)	39 (1.99)	< 0.001
MetS (NCEP)	909 (49.21)	531 (28.56)	168 (8.64)	27 (1.38)	< 0.001
MetS (IDF)	668 (36.17)	366 (19.69)	111 (5.71)	11 (0.56)	< 0.001

Male Arm-to-leg fat-free mass ratio	Q1 (n = 1322)	Q2 (n = 1358)	Q3 (n = 1370)	Q4 (n = 1375)	*P* value^a^
Hypertension	289 (21.11)	315 (23.01)	322 (23.23)	283 (21.75)	0.487
Diabetes	125 (9.13)	122 (8.91)	112 (8.08)	98 (7.53)	0.414
Dyslipidemia	116 (8.47)	135 (9.86)	118 (8.51)	76 (5.84)	0.002
MetS (NCEP)	370 (27.03)	380 (27.76)	377 (27.20)	308 (23.67)	0.072
MetS (IDF)	276 (20.16)	245 (17.90)	228 (16.45)	166 (12.76)	< 0.001

Female Arm-to-leg fat-free mass ratio	Q1 (n = 1828)	Q2 (n = 1902)	Q3 (n = 1956)	Q4 (n = 1921)	*P* value^a^
Hypertension	188 (9.38)	291 (15.09)	422 (22.29)	538 (30.22)	< 0.001
Diabetes	65 (3.24)	102 (5.29)	142 (7.50)	174 (9.78)	< 0.001
Dyslipidemia	136 (6.78)	180 (9.33)	193 (10.20)	224 (12.58)	< 0.001
MetS (NCEP)	276 (13.77)	387 (20.06)	451 (23.82)	521 (29.27)	< 0.001
MetS (IDF)	210 (10.47)	286 (14.83)	324 (17.12)	336 (18.88)	< 0.001

Male Limbs-to-trunk fat-free mass ratio	Q1 (n = 1322)	Q2 (n = 1358)	Q3 (n = 1370)	Q4 (n = 1375)	*P* value^a^
Hypertension	504 (37.89)	317 (23.14)	250 (18.23)	138 (10.19)	< 0.001
Diabetes	250 (18.80)	122 (8.91)	60 (4.38)	25 (1.85)	< 0.001
Dyslipidemia	162 (12.18)	129 (9.42)	91 (6.64)	63 (4.65)	< 0.001
MetS (NCEP)	518 (38.95)	378 (27.59)	318 (23.19)	221 (16.32)	< 0.001
MetS (IDF)	340 (25.56)	223 (16.28)	197 (14.37)	155 (11.45)	< 0.001

Female Limbs-to-trunk fat-free mass ratio	Q1 (n = 1828)	Q2 (n = 1902)	Q3 (n = 1956)	Q4 (n = 1921)	*P* value^a^
Hypertension	518 (28.34)	377 (19.82)	328 (16.77)	216 (11.24)	< 0.001
Diabetes	209 (11.43)	137 (7.20)	77 (3.94)	60 (3.12)	< 0.001
Dyslipidemia	226 (12.36)	214 (11.25)	170 (8.69)	123 (6.40)	< 0.001
MetS (NCEP)	566 (30.96)	410 (21.56)	372 (19.02)	287 (14.94)	< 0.001
MetS (IDF)	409 (22.37)	289 (15.19)	271 (13.85)	187 (9.73)	< 0.001

MetS, metabolic syndrome; NCEP, 2005 National Cholesterol Education Program Adult Treatment Panel III; IDF, 2006 International Diabetes Federation.

Data are presented as numbers (%).

^a^*P* value from χ^2^ test for categorical variables, comparing differences among four groups.

### Arm-to-leg fat mass ratio and cardiovascular disease risk factors

The ORs for CVD-RF according to the quartile of the arm-to-leg FM ratio are shown in Table 3. The ORs for HTN (men: OR 2.16, 95% confidence interval [CI] 1.62–2.88; women: OR 3.76, 95% CI 2.83–4.99), DM (men: OR 7.04, 95% CI 4.22–11.74; women: OR 10.57, 95% CI 5.80–19.26), DL (men: OR 3.09, 95% CI 2.05–4.68; women: OR 6.47, 95% CI 3.99–10.50), and MetS (men-NCEP: OR 4.47, 95% CI 3.41–5.86; men-IDF: OR 3.63, 95% CI 2.61–5.05; women-NCEP: OR 8.73, 95% CI 6.38–11.95; women-IDF: OR 9.42, 95% CI 6.41–13.86) were significantly higher in Q4 than in Q1 in both men and women after adjustment for age, TEI, WIBW, smoking, alcohol drinking, PA, education, AMHI, and survey year. The ORs significantly increased as the quartile increased from Q1 to Q4 (all *p* for trend < 0.001).

**Table 3:** Odds ratios for cardiovascular disease risk factors according to arm-to-leg fat mass ratio quartile.

Male	Arm-to-leg fat mass ratio quartile	Crude	Age-adjusted	Multivariable^a^
Hypertension	Q1	Reference	Reference	Reference
	Q2	2.29 (1.74–3.01)	1.57 (1.17–2.1)	1.58 (1.17–2.12)
	Q3	3.65 (2.83–4.72)	2.07 (1.56–2.74)	2.06 (1.55–2.73)
	Q4	4.57 (3.54–5.91)	2.15 (1.62–2.85)	2.16 (1.62–2.88)
	*p* for trend^b^	< 0.001	< 0.001	< 0.001
Diabetes	Q1	Reference	Reference	Reference
	Q2	3.44 (2.04–5.78)	2.4 (1.41–4.1)	2.48 (1.46–4.19)
	Q3	5.46 (3.3–9.05)	3.24 (1.93–5.43)	3.4 (2.03–5.68)
	Q4	12.73 (7.69–21.07)	6.66 (3.97–11.18)	7.04 (4.22–11.74)
	*p* for trend^b^	< 0.001	< 0.001	< 0.001
Dyslipidemia	Q1	Reference	Reference	Reference
	Q2	2.17 (1.45–3.26)	1.75 (1.15–2.66)	1.73 (1.13–2.64)
	Q3	3.62 (2.41–5.44)	2.59 (1.69–3.96)	2.5 (1.63–3.82)
	Q4	4.8 (3.26–7.07)	3.12 (2.06–4.71)	3.09 (2.05–4.68)
	*p* for trend^b^	< 0.001	< 0.001	< 0.001
MetS (NCEP)	Q1	Reference	Reference	Reference
	Q2	2.73 (2.1–3.54)	2.33 (1.79–3.04)	2.35 (1.8–3.07)
	Q3	4.8 (3.67–6.28)	3.74 (2.83–4.93)	3.65 (2.75–4.84)
	Q4	6.22 (4.82–8.03)	4.49 (3.45–5.86)	4.47 (3.41–5.86)
	*p* for trend^b^	< 0.001	< 0.001	< 0.001
MetS (IDF)	Q1	Reference	Reference	Reference
	Q2	2.49 (1.82–3.42)	2.2 (1.59–3.04)	2.24 (1.62–3.09)
	Q3	4.38 (3.18–6.03)	3.58 (2.57–5)	3.51 (2.51–4.91)
	Q4	4.71 (3.44–6.43)	3.62 (2.61–5.03)	3.63 (2.61–5.05)
	*p* for trend^b^	< 0.001	< 0.001	< 0.001

Female	Arm-to-leg fat mass ratio quartile	Crude	Age-adjusted	Multivariable^a^
Hypertension	Q1	Reference	Reference	Reference
	Q2	3.01 (2.24–4.05)	1.79 (1.28–2.5)	1.67 (1.19–2.33)
	Q3	6.32 (4.82–8.28)	2.61 (1.93–3.52)	2.38 (1.76–3.22)
	Q4	14.33 (11.03–18.6)	4.13 (3.09–5.5)	3.76 (2.83–4.99)
	*p* for trend^b^	< 0.001	< 0.001	< 0.001
Diabetes	Q1	Reference	Reference	Reference
	Q2	3.3 (1.65–6.62)	2.22 (1.12–4.39)	2.2 (1.11–4.36)
	Q3	8.2 (4.32–15.56)	4.11 (2.19–7.71)	3.98 (2.12–7.49)
	Q4	28.14 (15.34–51.63)	10.96 (6.02–19.95)	10.57 (5.8–19.26)
	*p* for trend^b^	< 0.001	< 0.001	< 0.001
Dyslipidemia	Q1	Reference	Reference	Reference
	Q2	3.76 (2.44–5.8)	2.7 (1.74–4.2)	2.49 (1.59–3.9)
	Q3	7.43 (4.85–11.38)	4.16 (2.67–6.48)	3.73 (2.37–5.86)
	Q4	15.83 (10.18–24.6)	6.91 (4.3–11.1)	6.47 (3.99–10.5)
	*p* for trend^b^	< 0.001	< 0.001	< 0.001
MetS (NCEP)	Q1	Reference	Reference	Reference
	Q2	3.23 (2.38–4.39)	2.29 (1.68–3.14)	2.26 (1.64–3.11)
	Q3	8.33 (6.22–11.16)	4.71 (3.49–6.36)	4.4 (3.25–5.96)
	Q4	21.05 (15.68–28.26)	9.25 (6.79–12.59)	8.73 (6.38–11.95)
	*p* for trend^b^	< 0.001	< 0.001	< 0.001
MetS (IDF)	Q1	Reference	Reference	Reference
	Q2	3.1 (2.07–4.64)	2.34 (1.56–3.51)	2.28 (1.51–3.44)
	Q3	8.98 (6.11–13.2)	5.57 (3.77–8.24)	5.12 (3.46–7.59)
	Q4	20.38 (14.04–29.58)	10.19 (6.94–14.96)	9.42 (6.41–13.86)
	*p* for trend^b^	< 0.001	< 0.001	< 0.001

Data are presented as odds ratio (95% confidence interval).

MetS, metabolic syndrome; NCEP, 2005 National Cholesterol Education Program Adult Treatment Panel III; IDF, 2006 International Diabetes Federation.

^a^Multivariable model adjusted for age, energy intake, water intake per body weight, smoking, alcohol drinking, physical activity, education, income, and survey year.

^b^Test for linear trend across quartile of arm-to-leg fat mass ratio.

### Limbs-to-trunk fat mass ratio and cardiovascular disease risk factors

The ORs for CVD-RF according to the quartile of the limbs-to-trunk FM ratio are shown in Table 4. The ORs for HTN (men: OR 0.25, 95% CI 0.19–0.33; women: OR 0.14, 95% CI 0.10–0.21), DM (men: OR 0.12, 95% CI 0.07–0.21; women: OR 0.12, 95% CI 0.06–0.23), DL (men: OR 0.17, 95% CI 0.10–0.27; women: OR 0.20, 95% CI 0.13–0.31), and MetS (men-NCEP: OR 0.06, 95% CI 0.04–0.08; men-IDF: OR 0.04, 95% CI 0.02–0.07; women-NCEP: OR 0.02, 95% CI 0.01–0.04; women-IDF: OR 0.02, 95% CI 0.01–0.03) were significantly lower in Q4 than in Q1 in both men and women after adjustment for age, TEI, WIBW, smoking, alcohol drinking, PA, education, AMHI, and survey year. The ORs significantly decreased as the quartile increased from Q1 to Q4 (all *p* for trend < 0.001).

**Table 4:** Odds ratios for cardiovascular disease risk factors according to limbs-to-trunk fat mass ratio quartile.

Male	Limbs-to-trunk fat mass ratio quartile	Crude	Age-adjusted	Multivariable^a^
Hypertension	Q1	Reference	Reference	Reference
	Q2	0.56 (0.46–0.68)	0.7 (0.56–0.86)	0.69 (0.56–0.86)
	Q3	0.26 (0.21–0.32)	0.4 (0.32–0.51)	0.41 (0.32–0.52)
	Q4	0.12 (0.09–0.15)	0.24 (0.19–0.32)	0.25 (0.19–0.33)
	*p* for trend^b^	< 0.001	< 0.001	< 0.001
Diabetes	Q1	Reference	Reference	Reference
	Q2	0.43 (0.32–0.57)	0.51 (0.38–0.69)	0.49 (0.37–0.66)
	Q3	0.2 (0.14–0.28)	0.3 (0.21–0.42)	0.28 (0.19–0.4)
	Q4	0.07 (0.04–0.12)	0.14 (0.08–0.23)	0.12 (0.07–0.21)
	*p* for trend^b^	< 0.001	< 0.001	< 0.001
Dyslipidemia	Q1	Reference	Reference	Reference
	Q2	0.58 (0.45–0.76)	0.65 (0.49–0.86)	0.67 (0.51–0.88)
	Q3	0.26 (0.19–0.37)	0.34 (0.24–0.48)	0.36 (0.25–0.51)
	Q4	0.1 (0.06–0.16)	0.15 (0.09–0.24)	0.17 (0.1–0.27)
	*p* for trend^b^	< 0.001	< 0.001	< 0.001
MetS (NCEP)	Q1	Reference	Reference	Reference
	Q2	0.49 (0.41–0.6)	0.53 (0.43–0.64)	0.52 (0.43–0.63)
	Q3	0.22 (0.17–0.27)	0.25 (0.2–0.31)	0.25 (0.2–0.31)
	Q4	0.04 (0.03–0.06)	0.06 (0.04–0.08)	0.06 (0.04–0.08)
	*p* for trend^b^	< 0.001	< 0.001	< 0.001
MetS (IDF)	Q1	Reference	Reference	Reference
	Q2	0.51 (0.41–0.63)	0.52 (0.42–0.65)	0.52 (0.41–0.65)
	Q3	0.22 (0.17–0.28)	0.23 (0.18–0.3)	0.23 (0.18–0.3)
	Q4	0.04 (0.02–0.06)	0.04 (0.02–0.07)	0.04 (0.02–0.07)
	*p* for trend^b^	< 0.001	< 0.001	< 0.001

Female	Limbs-to-trunk fat mass ratio quartile	Crude	Age-adjusted	Multivariable^a^
Hypertension	Q1	Reference	Reference	Reference
	Q2	0.48 (0.41–0.56)	0.66 (0.55–0.8)	0.66 (0.55–0.79)
	Q3	0.15 (0.12–0.18)	0.33 (0.26–0.43)	0.34 (0.26–0.43)
	Q4	0.03 (0.02–0.04)	0.13 (0.09–0.19)	0.14 (0.1–0.21)
	*p* for trend^b^	< 0.001	< 0.001	< 0.001
Diabetes	Q1	Reference	Reference	Reference
	Q2	0.4 (0.31–0.52)	0.51 (0.39–0.66)	0.49 (0.38–0.64)
	Q3	0.13 (0.09–0.19)	0.24 (0.16–0.36)	0.23 (0.16–0.35)
	Q4	0.04 (0.02–0.07)	0.12 (0.06–0.22)	0.12 (0.06–0.23)
	*p* for trend^b^	< 0.001	< 0.001	< 0.001
Dyslipidemia	Q1	Reference	Reference	Reference
	Q2	0.57 (0.45–0.72)	0.71 (0.55–0.91)	0.72 (0.57–0.93)
	Q3	0.22 (0.16–0.28)	0.37 (0.28–0.5)	0.38 (0.29–0.51)
	Q4	0.07 (0.05–0.1)	0.17 (0.11–0.27)	0.2 (0.13–0.31)
	*p* for trend^b^	< 0.001	< 0.001	< 0.001
MetS (NCEP)	Q1	Reference	Reference	Reference
	Q2	0.42 (0.36–0.49)	0.5 (0.42–0.58)	0.49 (0.41–0.58)
	Q3	0.09 (0.07–0.11)	0.14 (0.11–0.17)	0.14 (0.11–0.18)
	Q4	0.01 (0.01–0.02)	0.02 (0.01–0.04)	0.02 (0.01–0.04)
	*p* for trend^b^	< 0.001	< 0.001	< 0.001
MetS (IDF)	Q1	Reference	Reference	Reference
	Q2	0.45 (0.38–0.54)	0.52 (0.44–0.63)	0.52 (0.43–0.62)
	Q3	0.1 (0.08–0.14)	0.14 (0.11–0.19)	0.15 (0.11–0.2)
	Q4	0.01 (0–0.02)	0.01 (0.01–0.03)	0.02 (0.01–0.03)
	*p* for trend^b^	< 0.001	< 0.001	< 0.001

Data are presented as odds ratio (95% confidence interval).

MetS, metabolic syndrome; NCEP, 2005 National Cholesterol Education Program Adult Treatment Panel III; IDF, 2006 International Diabetes Federation.

^a^Multivariable model adjusted for age, energy intake, water intake per body weight, smoking, alcohol drinking, physical activity, education, income, and survey year.

^b^Test for linear trend across quartile of limbs-to-trunk fat mass ratio.

### Arm-to-leg fat-free mass ratio and cardiovascular disease risk factors

The ORs for CVD-RF according to the quartile of the arm-to-leg FFM ratio are shown in Table 5. The ORs for MetS (men-NCEP: OR 0.75, 95% CI 0.59–0.94; men-IDF: OR 0.52, 95% CI 0.39–0.7; women-NCEP: OR 0.73, 95% CI 0.58–0.92; women-IDF: OR 0.61, 95% CI 0.47–0.79) were significantly lower in Q4 than in Q1 in both men and women after adjustment for age, TEI, WIBW, smoking, alcohol drinking, PA, education, AMHI, and survey year. The ORs significantly decreased as the quartile increased from Q1 to Q4 (all *p* for trend < 0.05).

**Table 5:** Odds ratios for cardiovascular disease risk factors according to arm-to-leg fat-free mass ratio quartile.

Male	Arm-to-leg fat-free mass ratio quartile	Crude	Age-adjusted	Multivariable^a^
Hypertension	Q1	Reference	Reference	Reference
	Q2	1.26 (1.01–1.57)	1.02 (0.8–1.3)	1.01 (0.79–1.29)
	Q3	1.32 (1.06–1.64)	1.05 (0.82–1.36)	1.06 (0.81–1.39)
	Q4	1.19 (0.93–1.51)	0.91 (0.7–1.18)	0.93 (0.71–1.22)
	*p* for trend^b^	0.123	0.537	0.710
Diabetes	Q1	Reference	Reference	Reference
	Q2	1.06 (0.78–1.44)	0.87 (0.64–1.18)	0.85 (0.62–1.17)
	Q3	1.13 (0.82–1.55)	0.93 (0.67–1.29)	0.91 (0.66–1.25)
	Q4	1.11 (0.79–1.54)	0.9 (0.65–1.27)	0.84 (0.59–1.21)
	*p* for trend^b^	0.480	0.676	0.447
Dyslipidemia	Q1	Reference	Reference	Reference
	Q2	1.35 (0.98–1.85)	1.18 (0.85–1.64)	1.2 (0.86–1.67)
	Q3	1.22 (0.88–1.69)	1.06 (0.75–1.48)	1.11 (0.79–1.57)
	Q4	0.81 (0.57–1.14)	0.68 (0.47–0.97)	0.72 (0.49–1.04)
	*p* for trend^b^	0.230	0.024	0.085
MetS (NCEP)	Q1	Reference	Reference	Reference
	Q2	1.02 (0.83–1.25)	0.89 (0.72–1.09)	0.85 (0.69–1.05)
	Q3	0.98 (0.79–1.21)	0.84 (0.67–1.04)	0.8 (0.65–1)
	Q4	0.96 (0.77–1.2)	0.8 (0.64–0.99)	0.75 (0.59–0.94)
	*p* for trend^b^	0.679	0.033	0.011
MetS (IDF)	Q1	Reference	Reference	Reference
	Q2	0.87 (0.69–1.1)	0.78 (0.61–0.98)	0.74 (0.58–0.94)
	Q3	0.77 (0.61–0.98)	0.68 (0.54–0.86)	0.66 (0.51–0.84)
	Q4	0.65 (0.5–0.86)	0.55 (0.42–0.73)	0.52 (0.39–0.7)
	*p* for trend^b^	0.001	< 0.001	< 0.001

Female	Arm-to-leg fat-free mass ratio quartile	Crude	Age-adjusted	Multivariable^a^
Hypertension	Q1	Reference	Reference	Reference
	Q2	1.83 (1.44–2.33)	1.02 (0.78–1.34)	0.97 (0.74–1.28)
	Q3	3.14 (2.44–4.05)	1.29 (0.98–1.71)	1.17 (0.88–1.55)
	Q4	5.02 (3.95–6.38)	1.16 (0.88–1.54)	1.03 (0.78–1.36)
	*p* for trend^b^	< 0.001	0.140	0.611
Diabetes	Q1	Reference	Reference	Reference
	Q2	1.94 (1.33–2.84)	1.25 (0.84–1.85)	1.16 (0.78–1.73)
	Q3	2.79 (1.91–4.06)	1.37 (0.93–2.01)	1.2 (0.81–1.77)
	Q4	3.77 (2.67–5.32)	1.2 (0.81–1.78)	1.01 (0.68–1.5)
	*p* for trend^b^	< 0.001	0.434	0.852
Dyslipidemia	Q1	Reference	Reference	Reference
	Q2	1.66 (1.27–2.17)	1.12 (0.85–1.49)	1.07 (0.81–1.42)
	Q3	1.75 (1.31–2.35)	0.92 (0.67–1.26)	0.86 (0.63–1.17)
	Q4	2.77 (2.11–3.63)	0.99 (0.72–1.37)	0.96 (0.71–1.31)
	*p* for trend^b^	< 0.001	0.639	0.503
MetS (NCEP)	Q1	Reference	Reference	Reference
	Q2	1.61 (1.32–1.97)	1.06 (0.84–1.33)	1 (0.8–1.26)
	Q3	2 (1.64–2.44)	1 (0.81–1.24)	0.9 (0.73–1.12)
	Q4	2.64 (2.16–3.23)	0.84 (0.67–1.06)	0.73 (0.58–0.92)
	*p* for trend^b^	< 0.001	0.086	0.003
MetS (IDF)	Q1	Reference	Reference	Reference
	Q2	1.48 (1.18–1.85)	1.01 (0.79–1.28)	0.94 (0.74–1.2)
	Q3	1.82 (1.45–2.27)	0.98 (0.78–1.23)	0.84 (0.66–1.06)
	Q4	2.09 (1.65–2.65)	0.75 (0.59–0.97)	0.61 (0.47–0.79)
	*p* for trend^b^	< 0.001	0.020	< 0.001

Data are presented as odds ratio (95% confidence interval).

MetS, metabolic syndrome; NCEP, 2005 National Cholesterol Education Program Adult Treatment Panel III; IDF, 2006 International Diabetes Federation.

^a^Multivariable model adjusted for age, energy intake, water intake per body weight, smoking, alcohol drinking, physical activity, education, income, and survey year.

^b^Test for linear trend across quartile of arm-to-leg fat-free mass ratio.

### Limbs-to-trunk fat-free mass ratio and cardiovascular disease risk factors

The ORs for CVD-RF according to quartile of the limbs-to-trunk FFM ratio are shown in Table 6. The ORs for HTN (men: OR 0.46, 95% CI 0.35–0.60; women: OR 0.55, 95% CI 0.44–0.69), DM (men: OR 0.19, 95% CI 0.11–0.31; women: OR 0.46, 95% CI 0.30–0.70), DL (men: OR 0.60, 95% CI 0.39–0.91; women: Crude OR 0.45, 95% CI 0.34–0.60), and MetS (men-NCEP: OR 0.39, 95% CI 0.31–0.50; men-IDF: OR 0.43, 95% CI 0.33–0.57; women-NCEP: OR 0.62, 95% CI 0.50–0.78; women-IDF: OR 0.62, 95% CI 0.49–0.80) were significantly lower in Q4 than in Q1 in both men and women after adjustment for age, TEI, WIBW, smoking, alcohol drinking, PA, education, AMHI, and survey year. The ORs significantly decreased as the quartile increased from Q1 to Q4 (all *p* for trend < 0.001).

**Table 6:** Odds ratios for cardiovascular disease risk factors according to limbs-to-trunk fat-free mass ratio quartile.

Male	Limbs-to-trunk fat-free mass ratio quartile	Crude	Age-adjusted	Multivariable^a^
Hypertension	Q1	Reference	Reference	Reference
	Q2	0.48 (0.39–0.59)	0.67 (0.54–0.84)	0.66 (0.52–0.82)
	Q3	0.34 (0.28–0.42)	0.64 (0.51–0.81)	0.63 (0.5–0.81)
	Q4	0.17 (0.14–0.22)	0.48 (0.37–0.63)	0.46 (0.35–0.6)
	*p* for trend^b^	< 0.001	< 0.001	< 0.001
Diabetes	Q1	Reference	Reference	Reference
	Q2	0.47 (0.35–0.63)	0.63 (0.46–0.86)	0.66 (0.48–0.9)
	Q3	0.19 (0.13–0.27)	0.32 (0.22–0.45)	0.32 (0.23–0.46)
	Q4	0.08 (0.05–0.12)	0.18 (0.11–0.3)	0.19 (0.11–0.31)
	*p* for trend^b^	< 0.001	< 0.001	< 0.001
Dyslipidemia	Q1	Reference	Reference	Reference
	Q2	0.69 (0.51–0.92)	0.85 (0.63–1.15)	0.85 (0.63–1.16)
	Q3	0.45 (0.33–0.62)	0.65 (0.46–0.93)	0.67 (0.47–0.95)
	Q4	0.32 (0.22–0.47)	0.58 (0.38–0.89)	0.6 (0.39–0.91)
	*p* for trend^b^	< 0.001	0.005	0.008
MetS (NCEP)	Q1	Reference	Reference	Reference
	Q2	0.57 (0.47–0.7)	0.67 (0.55–0.83)	0.67 (0.54–0.82)
	Q3	0.43 (0.35–0.54)	0.58 (0.46–0.72)	0.58 (0.46–0.72)
	Q4	0.25 (0.2–0.31)	0.39 (0.31–0.5)	0.39 (0.31–0.5)
	*p* for trend^b^	< 0.001	< 0.001	< 0.001
MetS (IDF)	Q1	Reference	Reference	Reference
	Q2	0.52 (0.42–0.66)	0.6 (0.47–0.75)	0.58 (0.46–0.74)
	Q3	0.43 (0.34–0.54)	0.54 (0.42–0.68)	0.53 (0.42–0.68)
	Q4	0.31 (0.24–0.4)	0.44 (0.34–0.58)	0.43 (0.33–0.57)
	*p* for trend^b^	< 0.001	< 0.001	< 0.001

Female	Limbs-to-trunk fat-free mass ratio quartile	Crude	Age-adjusted	Multivariable^a^
Hypertension	Q1	Reference	Reference	Reference
	Q2	0.64 (0.53–0.78)	0.79 (0.64–0.99)	0.78 (0.63–0.98)
	Q3	0.5 (0.41–0.6)	0.81 (0.65–1.02)	0.81 (0.64–1.01)
	Q4	0.28 (0.22–0.35)	0.55 (0.43–0.69)	0.55 (0.44–0.69)
	*p* for trend^b^	< 0.001	< 0.001	< 0.001
Diabetes	Q1	Reference	Reference	Reference
	Q2	0.67 (0.5–0.89)	0.82 (0.6–1.1)	0.82 (0.61–1.12)
	Q3	0.32 (0.23–0.44)	0.47 (0.34–0.66)	0.47 (0.34–0.66)
	Q4	0.25 (0.17–0.37)	0.46 (0.31–0.68)	0.46 (0.3–0.7)
	*p* for trend^b^	< 0.001	< 0.001	< 0.001
Dyslipidemia	Q1	Reference	Reference	Reference
	Q2	0.81 (0.64–1.03)	0.99 (0.76–1.28)	0.96 (0.74–1.24)
	Q3	0.64 (0.5–0.83)	0.94 (0.72–1.24)	0.94 (0.71–1.23)
	Q4	0.45 (0.34–0.6)	0.79 (0.58–1.08)	0.8 (0.58–1.1)
	*p* for trend^b^	< 0.001	0.154	0.181
MetS (NCEP)	Q1	Reference	Reference	Reference
	Q2	0.57 (0.47–0.68)	0.65 (0.53–0.79)	0.64 (0.52–0.79)
	Q3	0.48 (0.41–0.57)	0.69 (0.57–0.83)	0.69 (0.57–0.83)
	Q4	0.36 (0.3–0.44)	0.63 (0.51–0.79)	0.62 (0.5–0.78)
	*p* for trend^b^	< 0.001	< 0.001	< 0.001
MetS (IDF)	Q1	Reference	Reference	Reference
	Q2	0.56 (0.46–0.69)	0.65 (0.53–0.8)	0.64 (0.51–0.79)
	Q3	0.51 (0.42–0.61)	0.7 (0.58–0.86)	0.71 (0.57–0.87)
	Q4	0.38 (0.3–0.48)	0.63 (0.49–0.8)	0.62 (0.49–0.8)
	*p* for trend^b^	< 0.001	< 0.001	< 0.001

Data are presented as odds ratio (95% confidence interval).

MetS, metabolic syndrome; NCEP, 2005 National Cholesterol Education Program Adult Treatment Panel III; IDF, 2006 International Diabetes Federation.

^a^Multivariable model adjusted for age, energy intake, water intake per body weight, smoking, alcohol drinking, physical activity, education, income, and survey year.

^b^Test for linear trend across quartile of limbs-to-trunk fat-free mass ratio.

### Fat mass or fat-free mass ratio between body parts according to age and body mass index

FM or FFM ratios between body parts according to age and BMI are shown in Supplementary Tables S1, S2. Compared to the group with BMI < 25, the arm-to-leg FM ratio was relatively higher (*p* < 0.001), and the arm-to-leg FFM ratio was also higher (*p* = 0.004) in the group with BMI ≥ 25. The limbs-to-trunk FM ratio was relatively lower (*p* < 0.001) in the group with BMI ≥ 25 than in the group with BMI < 25.

As age increased, the arm-to-leg FM ratio increased (*p* < 0.001), and the arm-to-leg FFM ratio also increased (*p* < 0.001). The limbs-to-trunk FM ratio decreased (*p* < 0.001), and limbs-to-trunk FFM ratio also decreased (*p* < 0.001) as age increased.

### Regional fat-to-fat-free mass ratio according to age and body mass index

Regional fat-to-fat-free mass ratios according to age and BMI are shown in Supplementary Tables S3, S4. Compared to the group with BMI < 25, the fat-to-fat-free mass ratio was higher in the arms, legs, and trunk in the group with BMI ≥ 25 (all *p* < 0.001). In other words, as BMI increased, FM increased at a higher rate than FFM in any part of the body.

As age increased, the fat-to-fat-free mass ratio in the arms and trunk increased (all *p* < 0.001), but the fat-to-fat-free mass ratio in the legs decreased (*p* < 0.001). In other words, with age, the proportion of FM increased in the arms and trunk, but the proportion of FM decreased in the legs.

## Discussion

This study analyzed data from the KNHANES to understand the association between FM or FFM ratios in the arms, legs, and trunk, and the prevalence of CVD-RF. The results of the present study showed that a higher FM ratio in the arms to legs was associated with a higher prevalence of CVD-RF, and a higher FM or FFM ratio in the limbs to trunk was associated with lower prevalence of CVD-RF, and a higher FFM ratio in the arms to legs was associated with lower prevalence of CVD-RF.

In previous studies, excessive FM in the legs had a positive effect on metabolic and cardiovascular risk^12–14^. This seems to be because gluteofemoral fat has a strong lipoprotein lipase activity and weak hormone-sensitive lipase activity, lowering low-density lipoprotein cholesterol and TG, and increasing HDL-C^24^. There are no previous studies on the relationship between the arm-to-leg FM ratio and the risk of CVD, but there are some studies that have analyzed the independent effects of FM in the arms, legs, and trunk. In the study that analyzed the risk of CVD according to FM distribution in children and adolescents, FM in the trunk had a positive correlation with low HDL-C, high TG, insulin resistance, and high C-reactive protein, while FM in the leg showed a negative correlation. There was no significant relationship in FM in the arm^14^. In other studies that analyzed the independent relationship between FM in the arms, legs, and trunk and the risk of CVD in adults, FM in the trunk was associated with lipid profile deterioration, and FM in the leg was associated with lipid profile improvement. There was no association between FM in the arm and lipid profile^25^. However, our results demonstrate that the higher the FM ratio in the arms to the legs, the higher the prevalence of CVD-RF. The present study also confirmed that the FM ratio of the arms increases compared to that of the legs with increasing age and BMI (Supplementary Tables S1, S2) and the proportion of FM (fat-to-fat-free mass ratio) increases in the arms and decreases in the legs with increasing age (Supplementary Table S4). Previously, our research group has reported that a high total fat-to-muscle ratio is significantly associated with the prevalence of MetS and insulin resistance^26^. Therefore, taking these factors into account, further studies are required to verify the correlation between FM in the arms and the risk of CVD and the correlation between the arm-to-leg FM ratio and the risk of CVD.

The finding that the higher the FM in the trunk than in the limbs and the lower FFM in the limbs than in the trunk, the higher the prevalence of CVD-RF is consistent with the results of many previous studies showing that abdominal fat increases the risk of CVD^6,7,27^. Therefore, in patients with excessive abdominal fat, reducing body weight and abdominal fat through lifestyle modification on diet and physical activity may reduce the risk of CVD by improving insulin sensitivity and reducing TG, LDL, and BP. Moreover, in the previous study, the higher the total FFM, the lower the risk of MetS^28^. However, there are no previous studies comparing directly to the trunk and limb FFM with the risk of MetS and CVD. In a previous study that analyzed skeletal muscle distribution using magnetic resonance imaging, it was reported that the femoral muscles accounted for the largest proportion of total FFM and that the reduction of leg muscles contributed primarily to the reduction of muscle due to aging^29^. The present study also confirmed that the FM ratio of the trunk increases compared to that of the limbs with increasing age and BMI (Supplementary Tables S1, S2), the FFM ratio of the limbs decreases compared to that of the trunk with increasing age (Supplementary Table S2), and the proportion of FM (fat-to-fat-free mass ratio) increases in the trunk with increasing age (Supplementary Table S4). In addition, the muscle quality of each part of the body is affected not only by aging but also by factors such as physical activity^30^. Therefore, taking these factors into account, further research is needed on the relationship between the limbs-to-trunk FFM ratio and the risk of CVD.

The present study confirmed that the higher the arm-to-leg FFM ratio, the lower the prevalence of MetS. This may be related to the fact that glucose clearance is higher in the arm muscles than in the legs, regardless of insulin resistance, and insulin sensitivity is comparatively better preserved in the arm muscles, especially in patients with type 2 DM^31^. Although there are few studies that directly compared FFM in the arm and the risk of CVD, studies related to arm circumference, which is used as a surrogate marker for the arm muscle mass, indirectly report the relationship between FFM in the arm and a risk of CVD. One previous trial has shown that arm circumference was negatively correlated with all-cause mortality^32^, and another trial reported that arm circumference was negatively correlated with all-cause and CVD mortality^33^. However, there are studies that showed negative effects contrary to this finding. In the study that analyzed the relationship between arm circumference and the risk of CVD, the risk of CVD increased as the arm circumference increased^34^. Another study suggested that arm circumference had a significant positive association with MetS^35^. The inconsistent results between these previous studies may be due to the analysis of FFM without considering the ratio of arms to trunk or legs, or it may be an error caused by indirectly measuring arm FFM with arm circumference. The present study also confirmed that the proportion of FFM decreases in the arms with increasing age and BMI (Supplementary Tables S3, S4). Using arm circumference, it is difficult to distinguish between FM and FFM, and it is difficult to consider fat within muscle. Even if measured by DEXA, it is difficult to determine fat within muscle. Therefore, taking these factors into account, further studies are required on the relationship between the arm-to-leg FFM ratio and the risk of CVD.

This study is meaningful in that it used large-scale data from the representative KNHANES for 4 years. However, there are several limitations. First, the data based on surveys may have a recall bias. Second, because this is a cross-sectional study, it is not easy to prove a causal relationship to the risk of CVD. Third, although the analysis was conducted by adjusting for age, TEI, smoking, alcohol drinking, PA, education, AMHI, and survey year, potential confounding factors that were not considered cannot be ruled out. Finally, the FM and FFM were measured using DEXA, but it was difficult to accurately distinguish between subcutaneous fat, visceral fat, and fat within muscle.

## Conclusions

In conclusion, the association between FM or FFM ratios in the arms, legs, and trunk, and the prevalence of CVD-RF was analyzed. The higher the FM in the legs than in the arms, FFM in the arms than in the legs, and FM or FFM in the limbs than in the trunk, the lower the prevalence of CVD-RF. The findings of this study can be used as a guideline for improving body composition to reduce the prevalence of CVD-RF in patients with obesity or sarcopenic obesity.

## Supplementary Information

Supplementary Information.

## Data Availability

The datasets generated during and/or analysed during the current study are publicly available from the Korea National Health and Nutrition Examination Survey database (URLs: https://knhanes.kdca.go.kr/knhanes/sub03/sub03_02_05.do, https://knhanes.kdca.go.kr/knhanes/eng/index.do).
