# Supplementary material for: Relationship between arm-to-leg and limbs-to-trunk body composition ratio and cardiovascular disease risk factors
Source: Sci Rep. 2021 Aug 31;11:17414. doi: 10.1038/s41598-021-96874-8 (PMC8408188; doi:10.1038/s41598-021-96874-8)
Supplement: Supplementary file 1 — Supplementary Information. [file 41598_2021_96874_MOESM1_ESM.docx]

**Title:** Relationship between Arm-to-Leg and Limbs-to-Trunk Body Composition Ratio and Cardiovascular Disease Risk Factors

**Running Title:** Body Composition Ratio and Cardiovascular Disease Risk Factors

Sunmi Jung, Jihyun Park, Young-Gyun Seo^*^

Department of Family Medicine, Hallym University Sacred Heart Hospital, Anyang, Gyeonggi-do, Korea

^*^Corresponding Author: Young-Gyun Seo, MD. PhD.

Department of Family Medicine, Hallym University Sacred Heart Hospital

22, Gwanpyeong-ro 170beon-gil, Dongan-gu, Anyang-si, Gyeonggi-do 14068, Republic of Korea.

E-mail: yg035@daum.net; Phone: +82-31-380-3805; Fax: +82-31-380-1782

**Supplementary Table S1.** Body composition ratio according to body mass index

|  | **Total**  **(n = 13,032)** | **BMI < 25**  **(n = 8,957)** | **BMI ≥ 25**  **(n = 4,075)** | P value^a^ |
| --- | --- | --- | --- | --- |
| **Arm-to-leg fat mass ratio** | 0.37±0.001 | 0.35±0.001 | 0.40±0.002 | <0.001 |
| **Limbs-to-trunk fat mass ratio** | 0.87±0.004 | 0.94±0.004 | 0.73±0.004 | <0.001 |
| **Arm-to-leg fat-free mass ratio** | 0.32±0.0007 | 0.316±0.0008 | 0.319±0.0009 | 0.004 |
| **Limbs-to-trunk fat-free mass ratio** | 0.86±0.001 | 0.85±0.001 | 0.86±0.002 | 0.395 |

Abbreviations: BMI, Body mass index.

^a^P value from linear regression analysis, comparing differences between two groups.

**Supplementary Table S2.** Body composition ratio according to age

|  | **Total**  **(n = 13,032)** | **< 30s**  **(n = 1,693)** | **30s**  **(n = 2,624)** | **40s**  **(n = 2,558)** | **50s**  **(n = 2,431)** | **60s**  **(n = 2,195)** | **70s**  **(n = 1,345)** | **≥ 80s**  **(n = 186)** | p for trend^a^ |
| --- | --- | --- | --- | --- | --- | --- | --- | --- | --- |
| **Arm-to-leg fat mass ratio** | 0.37±0.001 | 0.31±0.002 | 0.34±0.002 | 0.37±0.002 | 0.40±0.002 | 0.42±0.003 | 0.41±0.003 | 0.39±0.007 | <0.001 |
| **Limbs-to-trunk fat mass ratio** | 0.87±0.004 | 1.06±0.009 | 0.94±0.006 | 0.84±0.006 | 0.76±0.004 | 0.72±0.005 | 0.73±0.006 | 0.75±0.01 | <0.001 |
| **Arm-to-leg fat-free mass ratio** | 0.32±0.0007 | 0.30±0.001 | 0.31±0.001 | 0.32±0.001 | 0.33±0.001 | 0.33±0.001 | 0.33±0.001 | 0.32±0.003 | <0.001 |
| **Limbs-to-trunk fat-free mass ratio** | 0.86±0.001 | 0.89±0.003 | 0.87±0.002 | 0.85±0.002 | 0.84±0.002 | 0.83±0.002 | 0.82±0.003 | 0.81±0.008 | <0.001 |

^a^Test for linear trend across age.

**Supplementary Table S3.** Fat-to-fat-free mass ratio according to body mass index

|  | **Total**  **(n = 13,032)** | **BMI < 25**  **(n = 8,957)** | **BMI ≥ 25**  **(n = 4,075)** | P value^a^ |
| --- | --- | --- | --- | --- |
| **Arm fat-to-fat-free mass ratio** | 0.50±0.004 | 0.47±0.004 | 0.57±0.006 | <0.001 |
| **Leg fat-to-fat-free mass ratio** | 0.42±0.003 | 0.41±0.003 | 0.44±0.004 | <0.001 |
| **Limbs fat-to-fat-free mass ratio** | 0.44±0.003 | 0.42±0.003 | 0.47±0.005 | <0.001 |
| **Trunk fat-to-fat-free mass ratio** | 0.43±0.003 | 0.38±0.003 | 0.54±0.004 | <0.001 |

Abbreviations: BMI, Body mass index.

^a^P value from linear regression analysis, comparing differences between two groups.

**Supplementary Table S4.** Fat-to-fat-free mass ratio according to age

|  | **Total**  **(n = 13,032)** | **< 30s**  **(n = 1,693)** | **30s**  **(n = 2,624)** | **40s**  **(n = 2,558)** | **50s**  **(n = 2,431)** | **60s**  **(n = 2,195)** | **70s**  **(n = 1,345)** | **≥ 80s**  **(n = 186)** | p for trend^a^ |
| --- | --- | --- | --- | --- | --- | --- | --- | --- | --- |
| **Arm fat-to-fat-free mass ratio** | 0.50±0.004 | 0.48±0.008 | 0.49±0.006 | 0.50±0.006 | 0.51±0.007 | 0.52±0.007 | 0.53±0.009 | 0.51±0.02 | <0.001 |
| **Leg fat-to-fat-free mass ratio** | 0.42±0.003 | 0.44±0.007 | 0.43±0.005 | 0.42±0.005 | 0.40±0.005 | 0.40±0.005 | 0.41±0.007 | 0.42±0.02 | <0.001 |
| **Limbs fat-to-fat-free mass ratio** | 0.44±0.003 | 0.45±0.007 | 0.45±0.005 | 0.43±0.005 | 0.43±0.005 | 0.43±0.005 | 0.44±0.007 | 0.44±0.02 | 0.009 |
| **Trunk fat-to-fat-free mass ratio** | 0.43±0.003 | 0.38±0.005 | 0.41±0.004 | 0.43±0.004 | 0.46±0.005 | 0.49±0.005 | 0.49±0.008 | 0.49±0.02 | <0.001 |

^a^Test for linear trend across age.

**Supplementary Fig. S1.** Participants flowchart

Missing data (n = 4,343)

Inappropriate water intake per body weight (≥90 g/kg) (n = 5)

Inappropriate nutritional intake (<500 kcal/day or >5000 kcal/day) (n = 217)

A history of cancer diagnosis (n = 598)

Estimated glomerular filtration rate <30 mL/min/1.73m^2^ (n = 82)

Age <19 years (n = 1,919)

Inappropriate fasting duration before examination (>24 hours or <8 hours)

(n = 638)

No missing data

N = 13,032

Appropriate water intake per body weight

N = 17,375

Appropriate nutritional intake

N = 17,380

Appropriate fasting duration before examination

N = 17,597

No history of cancer diagnosis

N = 18,235

Estimated glomerular filtration rate ≥30 mL/min/1.73m^2^

N = 18,833

The 4th and 5th Korea National Health and Nutrition Examination Survey (2008-2011)

N = 20,834

Age ≥19 years

N = 18,915
